# Microbial Genetics and Clonal Dissemination of *Salmonella* *enterica* Serotype Javiana Isolated from Human Populations in Arkansas, USA

**DOI:** 10.3390/pathogens11101192

**Published:** 2022-10-16

**Authors:** Yasser M. Sanad, Nesreen H. Aljahdali, Bijay K. Khajanchi, Rajesh Nayak, Ashraf Khan, Steven L. Foley

**Affiliations:** 1 Division of Microbiology, National Center for Toxicological Research, U.S. Food and Drug Administration, Jefferson, AR 72079, USA; 2 Department of Agriculture, University of Arkansas at Pine Bluff, Pine Bluff, AR 71601, USA; 3 Biological Science Department, College of Science, King AbdulAziz University, Jeddah 21551, Saudi Arabia; 4 Regulatory Compliance and Risk Management, National Center for Toxicological Research, U.S. Food and Drug Administration, Jefferson, AR 72079, USA

**Keywords:** *Salmonella* Javiana, antimicrobial resistance, pulsed-field gel electrophoresis (PFGE), replicon typing, molecular typing, whole genome sequencing (WGS)

## Abstract

*Salmonella* is estimated to cause over a million infections and ~400 deaths annually in the U.S. *Salmonella enterica* serotype Javiana strains (n = 409) that predominantly originated from the State of Arkansas over a six-year period (2003 to 2008) were studied. This period coincided with a rapid rise in the incidence of *S.* Javiana infections in the U.S. Children under the age of 10 displayed the highest prevalence of *S*. Javiana infections, regardless of sex or year of detection. Antimicrobial susceptibility to 15 different antimicrobials was assessed and 92% (n = 375) were resistant to at least one of the antimicrobials. Approximately 89% of the isolates were resistant to sulfisoxazole alone and 3% (n = 11) were resistant to different antimicrobials, including gentamicin, ciprofloxacin or ceftiofur. The pulsed-field gel electrophoresis (PFGE) analyses assessed the genotypic diversity and distribution of *S*. Javiana strains using *Xba*I restriction. Nine major clusters were identified and isolates from each group were digested with the restriction enzyme *Avr*II. Isolates with identical profiles of *Xba*I and *Avr*II were found to be disseminated in human populations. These distinct “types” of *S*. Javiana were persistent in human populations for multiple years. A subset of isolates (n = 19) with unique resistance phenotypes underwent plasmid and incompatibility (Inc) type analyses and the isolates resistant to more than one antimicrobial harbored multiple plasmids (<3 to 165 kb). Furthermore, these strains possessed 14 virulence genes, including *pagC, cdtB,* and *iroN*. The whole genome sequences (WGS) of 18 isolates that mostly originated from Arkansas from 2003 to 2011 were compared with isolates collected from different areas in the U.S. in 1999, indicating the perseverance of *S*. Javiana in disseminating antimicrobial resistance and virulence genes.

## 1. Introduction

*Salmonella enterica* infections are a significant public health concern worldwide, with an estimated 1.35 million cases, 26,500 hospitalizations and ~420 deaths in the United States (U.S.) each year [1]. Human salmonellosis is typically associated with the consumption of contaminated foods, such as fresh and processed meat and poultry, eggs and fresh produce [2]. The most detected serotypes causing human infections in the U.S. are the serotypes Enteritidis, Typhimurium, Newport, Javiana and Heidelberg [3,4], leading to approximately 50% of reported human salmonellosis cases in 2009 [5]. Plant-associated serotypes, such as *S.* Javiana, are particularly relevant given increases in *Salmonella* outbreaks attributed to fruits or vegetables in the last several years [6,7]. Recently, an outbreak of *S.* Javiana originated from Tailor Cut Produce in New Jersey and involved 165 people from 14 states with illnesses occurring from November 2019 to January 2020 [8].

Furthermore, Javiana was among other *Salmonella* serovars recovered from surface water in the southeastern U.S. [9]. From 1996 to 2007, *S*. Javiana was the fifth most prevalent *Salmonella* serovar reported to the CDC before moving to fourth in 2008. Compared to 1996, the number of cases reported to the CDC increased by nearly 360% in 2015 to 2696 cases [6,10], demonstrating a substantial increase of this pathogen in the U.S. The incidence of *S*. Javiana increased significantly in the Foodborne Diseases Active Surveillance Network (FoodNet) sites by 167% from 1996–1998 to 2004 [11]. In 2015, *S.* Javiana accounted for 5.6% of all reported *Salmonella* cases to the CDC [4]. *S*. Javiana infections have been most commonly detected in the southeastern U.S. and have frequently been associated with amphibian species that correspond to these regions [12]. These authors also noted that this animal reservoir corresponds with the seasonality of *S*. Javiana-associated outbreaks that typically peak between July and September. The overall incidence of *Salmonella* infection has not changed since 2014-2016 owing to increases in infections caused by the serotypes Javiana, Thompson, and Infantis (50% increase), while the incidence of the serotypes Heidelberg and Typhimurium has significantly decreased [13]. There also appears to be some parallels between outbreaks, periods when rainfall levels were at their highest and the increased frequencies of frogs and toads that occurred during these times [12]. *S*. Javiana outbreaks have also been linked to tomatoes [14,15], cheese [16], and chicken sandwiches [17]. In 2003, a food handler was responsible for an outbreak of *S*. Javiana at a children’s hospital in Missouri that affected more than 600 people [18]. In 2004, the CDC and Pennsylvania Department of Health reported a multi-state (Pennsylvania, West Virginia, Virginia, Maryland and Ohio) outbreak of *S*. Javiana gastroenteritis associated with eating food purchased from gas station deli counters [14]. By examining the diversity and predominance of food products implicated in outbreaks of salmonellosis during 1998–2008, more than 50% of outbreaks caused by *S.* Javiana were attributed to eggs or poultry. *S.* Javiana represented 30% of ten outbreaks associated with fruits in general, and two of the ten leafy-vegetable-associated outbreaks [19]. 

It was believed for a long time that *S.* Javiana is one of the serotypes with limited geographical distribution because it was mainly associated with amphibians, an animal host that has the highest numbers in the southeastern U.S. Human infections did not seem to be associated with consuming a particular food; however, in the last few decades the range has expanded across Florida, along the Gulf Coast and up the Atlantic Coast [20]. However, since major outbreaks have been linked to contaminated produce from the southeast, scientists suggested that *S*. Javiana may have an environmental host that sometimes leads to contamination of fresh produce. Further, recent Javiana outbreaks have begun from the northern climates and spread among several northern states, which may indicate an expansion of the geographic range of its host. It is imperative to better understand how *S.* Javiana has emerged as a prominent human pathogen, and there are further needs to assess its threat to cause outbreaks. There is limited information available on the genetic and evolutionary characteristics of this pathogen, which could continue to be a dominant *Salmonella* serovar infecting humans. This study was conducted to evaluate the genetic and epidemiological characteristics of this historical set of pathogens from human clinical origins to help better our understanding of *Salmonella* to develop further efficient interventional and preventive strategies to limit infections. 

## 2. Results

### 2.1. Epidemiology and Demographics

**Figure 1:**
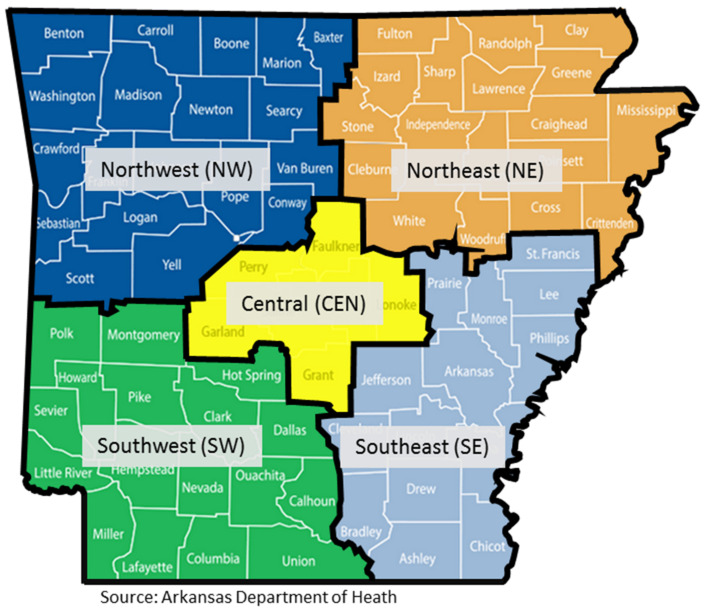
Geographical regions used for reporting the numbers of cases of *S.* Javiana. The regions are defined by the Arkansas Department of Health, the source of the isolates for the current study.

A total of 409 *S*. Javiana isolates collected from 2003 to 2008 were analyzed, 96% (394/409) of which originated from Arkansas, while the remaining 4% originated from other U.S. States (Table 1). Nearly 98% (386/394) of isolates collected in Arkansas originated from known regions within the State (Figure 1, Table 1), while specific geographical information was unavailable for the remaining eight isolates. Nearly 41 and 33% of the isolates were sampled from the central (CEN) and northeast (NE) regions, respectively, while the remaining 26% of the isolates originated from other regions (Table 1).

**Figure 2:**
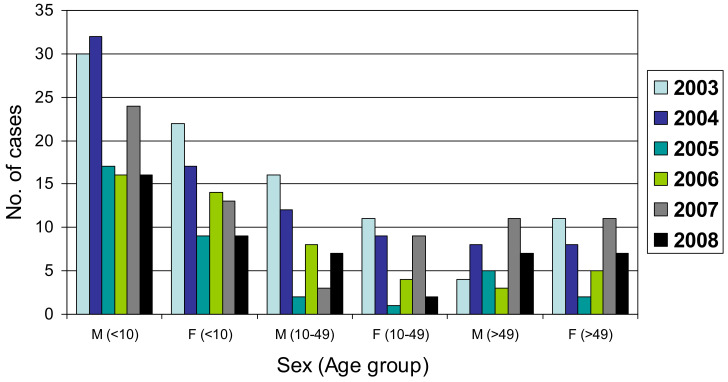
Distribution of *S.* Javiana cases based on age, sex and year of isolation.

Approximately 43% of the strains were isolated from female subjects (Table 2). Additionally, 54% of the bacterial strains were isolated from patients <10 years old. Approximately 48% of *S*. Javiana isolates were isolated from 2003 to 2004, while the frequency of isolation decreased and remained nearly flat from 2005 to 2008. It appeared that regardless of the year of detection, children under the age of 10 displayed the highest prevalence of infections (Figure 2).

### 2.2. Antimicrobial Susceptibility Testing

The 409 *S*. Javiana isolates in this study showed 11 antimicrobial susceptibility profiles (Table 3). Nearly 92% were resistant to at least one of the antimicrobials tested, with 89% resistant to sulfisoxazole (SUL). Additional resistance phenotypes included resistance to gentamicin (GEN), streptomycin (STR), chloramphenicol (CHL), tetracycline (TET), nalidixic acid (NAL), ciprofloxacin (CIP) or ceftiofur (TIO), with or without SUL resistance.

### 2.3. Distribution of Salmonella Javiana Genotypes

Analysis of the *XbaI* pulsed-field gel electrophoresis (PFGE) data revealed 116 distinct patterns. A group of isolates was considered to be a major cluster if it consisted of at least 2% of the study isolates (>8 isolates) with a 100% similarity index. A total of nine major clusters with identical DNA restriction profiles were identified (Supplemental Figure S1). The number of isolates within each cluster ranged from 9 to 48 isolates. There was a limited correlation between each cluster and the epidemiology of the bacterial isolates, suggesting that the bacterial populations were widely dispersed. 

**Figure 3:**
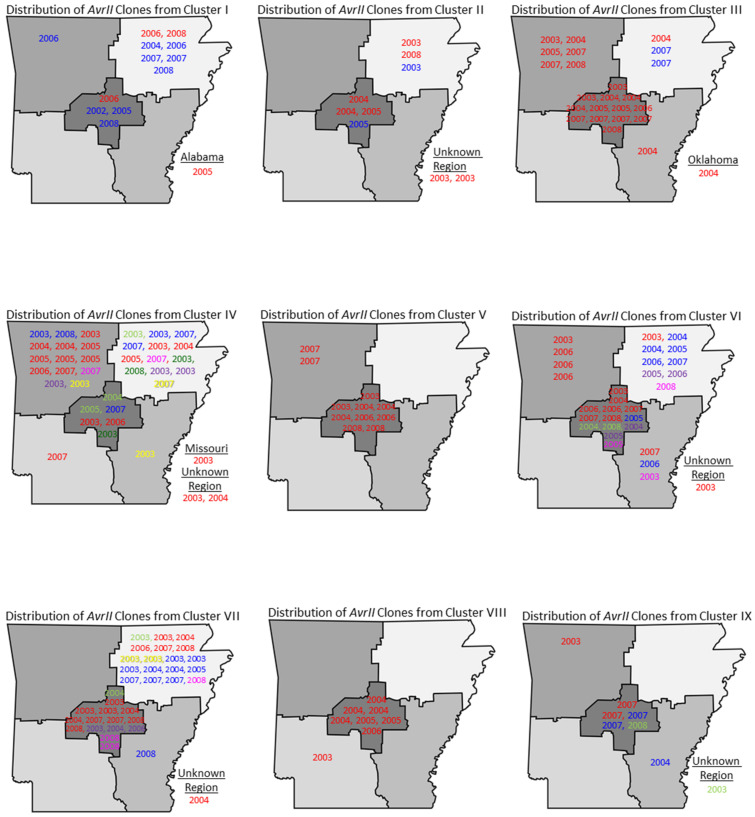
Geographical and temporal distribution of *S.* Javiana “types” based on *Xba*I and *Avr*II PFGE analyses. The nine clusters are defined based on identical *Xba*I PFGE profiles based on the analyses shown in Supplemental Figure S1. Among the clusters, isolates with common colors in each subfigure represent isolates with identical *Avr*II profiles (i.e., types) and the year indicates the year of isolation.

Isolates from within each cluster were subsequently digested with *AvrII* and analyzed by PFGE to determine the clonality within the clusters. Isolates that were identical in their DNA fingerprint profiles by both restriction enzymes (*XbaI* and *AvrII*) were termed “types”, or simply these isolates likely shared a common genetic lineage. Figure S1 highlights the clustering dendrograms for *XbaI* and *AvrII*-digested DNA fingerprints for *S*. Javiana. The colored circles in the *AvrII* clusters represent the identity of *S*. Javiana types within the respective clusters and correspond to colored dates shown in Figure 3 displaying the spatial and temporal distribution of types among human patients.

### 2.4. Plasmid Profiles

**Figure 4:**
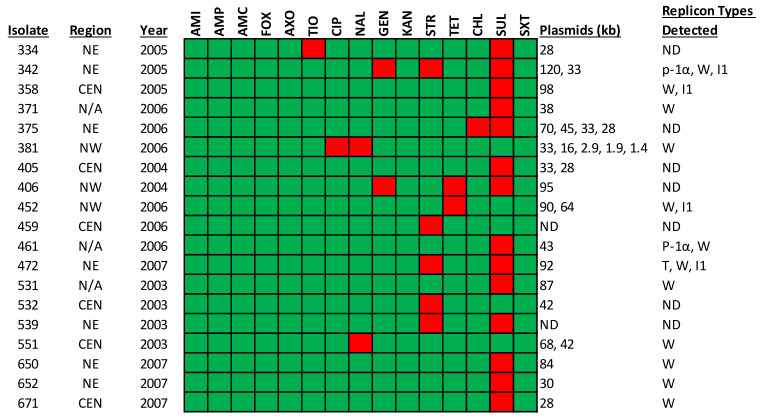
Antimicrobial susceptibility profiles and plasmid characterization of *S*. Javiana. In the figure, a green box represents susceptibility, and a red box indicates resistance to the corresponding antimicrobial. The regions are defined in Figure 1. The abbreviations for the antimicrobials are: amikacin (AMI), amoxicillin-clavulanic acid (AMC), ampicillin (AMP), cefoxitin (FOX), ceftiofur (TIO), ceftriaxone (AXO), ciprofloxacin (CIP), chloramphenicol (CHL), gentamicin (GEN), kanamycin (KAN), nalidixic acid (NAL), streptomycin (STR), sulfisoxazole (SUL), trimethoprim-sulfamethoxazole (SXT), and tetracycline (TET).

Nineteen *S*. Javiana isolates, including those that displayed a distinct antimicrobial resistance phenotype, were analyzed for plasmids (Figure 4). Twelve of nineteen (63%) isolates harbored at least one replicon type, while 42% of the isolates harbored two to four replicons (Figure 4). Four replicon types (p-1α, T, W or I1) were identified among the isolates. Plasmid replicon typing data indicated that 63 and 21% of the isolates harbored the replicon types W and I1, respectively. Two of the isolates had plasmids in IncP-1α, while one isolate had a plasmid, IncT (Figure 4).

### 2.5. Virulence Gene Profiles

**Figure 5:**
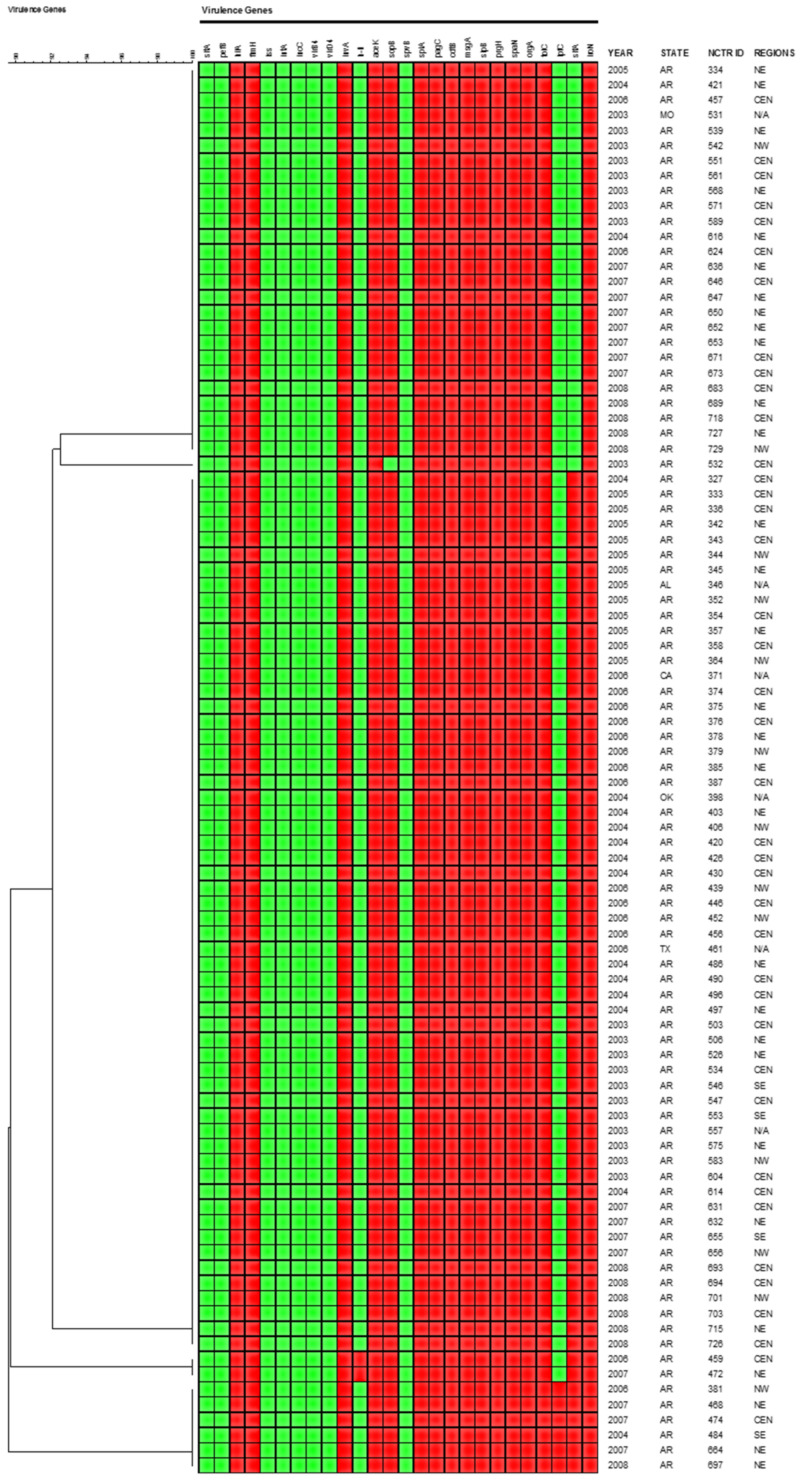
Virulence gene profiles of *S.* Javiana strains. A red box indicates the presence of the corresponding resistance gene by PCR detection and a green box indicates the absence of the gene based on PCR results. The dendrogram is based on the presence/absence of the different genes studied. The regions are defined in Figure 1.

Polymerase chain reaction (PCR) analysis was used to determine the presence of the virulence genes in 93 *S*. Javiana isolates were selected based on their different epidemiological and fingerprint results. The following genes were detected in all bacterial isolates: *hilA, fimH, invA, aceK, spiA, pagC, cdtB, msgA, sipB, prgH, tolC, spaN, orgA, tolC and iroN* (Figure 5). Nearly 73% of the isolates were positive for *sifA*, while 6% were positive for the *lpfC* gene. Most strains (98%) were negative for *h-li* with the exceptions of strains 459 and 472. Genes *sitA, pefB, iss, iutA, iucC, virB4, virD4* and *spvB* were absent in all isolates.

### 2.6. Whole Genome Sequence (WGS) Analysis

**Figure 6:**
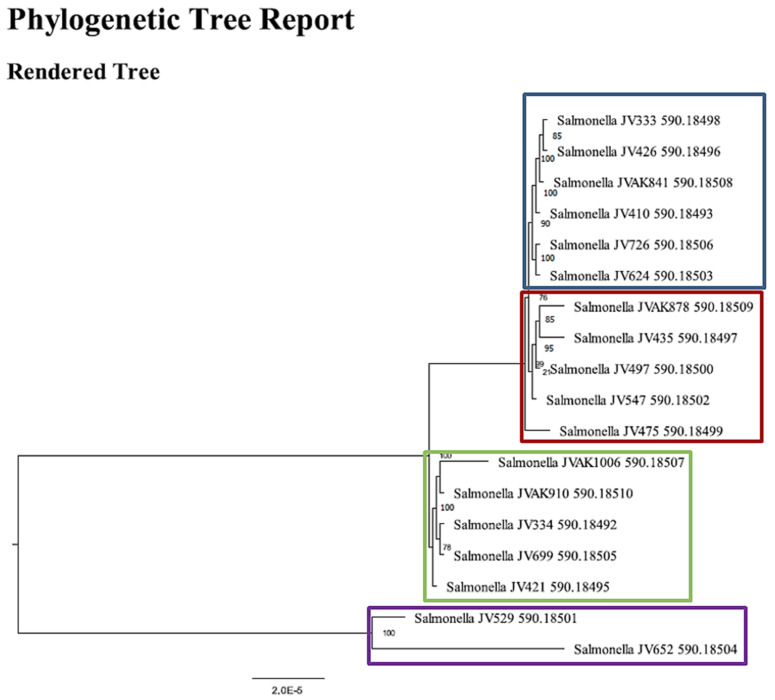
Phylogenetic tree for 18 *S*. Javiana. Phylogenetic analysis using PATRIC platform indicates some isolates clustered together based on the virulence genes and most of the isolates grouped together based on geographic region. The numbers on the scale present the percentage of genetic variation.

WGS, using PATRIC software to detect the virulence genes, revealed that 18 isolates had very similar virulence factor profiles, such as *sopAD2*, *fimYD*, *pagOD*, *sitA*, *iroB*, *pipABCD*, and *sspA*. Using SNP based on phylogenetic analyses, the genetic structure of *S*. Javiana isolates 333, 426, 841, 410, 726, and 624 clustered together, which shared a high degree of 138 virulence genes (Figure 6; blue box). Although most of the isolates had very similar virulence factor profiles, the strains 878, 435, 497, 547, and 475 grouped together because they carried the lipopolysaccharide 1,3-galactosyltransferase (*rfaI*) gene (Table 4; Figure 6; red box). Remarkably, the isolates of *S.* Javiana collected in 1999 clustered with the isolates of the current study from 2003–2011, which likely indicates that these isolates shared a high degree of genetic relatedness and carried similar virulence factors with other strains (Figure 6; Table 4 and Table 5).

WGS analyses, using ResFinder software to identify antimicrobial resistance genes, found that 17 strains (94%) contained *parC*, which in cases of certain mutations within the gene can contribute to resistance to nalidixic acid and ciprofloxacin (Table 5). Furthermore, the SNP-based evolutionary tree showed that the genetic structure of *S*. Javiana plays a significant role in grouping strains based on geographic region. To illustrate, the isolates 333, 426, and 841, originating from central (CEN) Arkansas, clustered together, as did isolates 726 and 624, collected from CEN Arkansas. Noticeably, isolate 410, collected from Michigan, clustered with the isolates originating from CEN Arkansas, which indicated these isolates shared very similar virulence factor profiles (Figure 6; blue box; Table 4 and Table 5). Additionally, isolates 1006, 910, 334, and 421, collected from northeast (NE) Arkansas, grouped together. Remarkably, isolate 699, originating from an unknown location within the U.S., clustered with the isolates collected from NE Arkansas (Table 4 and Table 5; Figure 6 green box). Similarly, the isolates 529 and 652, collected from NE Arkansas, grouped together (Table 4 and Table 5; Figure 6 purple box).

## 3. Discussion

This study evaluated the diversity and distribution of *S.* Javiana strains that were isolated from clinical cases from different locations, primarily from the State of Arkansas, during the years 2003 to 2008; a period where the incidence of *S.* Javiana infections in the U.S. increased from 0.41 to 0.70 cases per 100,000 people [4]. Put another way, in 2002, the year prior to the study period, there were 1201 laboratory-confirmed illnesses caused by *S.* Javiana in the U.S., however, during the study period, the numbers increased to 2329 in 2008 [5], the last year of the study period. The numbers of reported infections have remained relatively high in the years following the study period [4]. Studies have indicated that 70% of *Salmonella* outbreaks caused by *S.* Javiana were linked to plant-derived food products [19]. It was previously reported that *S.* Javiana is more prevalent in the southeast U.S. and is mainly associated with exposure to amphibians [12]. Boore et al. (2015) examined the epidemiologic characteristics of individual serotypes of *Salmonella enterica* in the period between 1996 to 2011 and reported that *S*. Javiana was most frequently detected in the southern U.S., which includes the State of Arkansas, with a coefficient of variation (CV 135%) [3]. 

There is also a seasonal variation in the numbers of infections caused by the serotype, with the most common months of infection being in the summer (July and August) and gradually declining throughout the fall for school-aged children and adults [20]. Interestingly, for pre-school children (under the age of five), the percentage of infections increases throughout the summer and peaks in September and October [20]. 

Our data indicated that children under the age of 10 were most likely to acquire *S.* Javiana infections regardless of sex or year of detection (Figure 2). *S.* Javiana’s association with amphibians and reptiles might also explain part of its temporal and geographic distribution [3,12]. Clarkson et al. (2010) reported that *S*. Javiana infections in the southeastern U.S. were associated with environmental factors such as well water and/or contact with animal reservoirs, such as reptiles or amphibians, with an adjusted odds ratio of 4•3 and 2•6, respectively [21]. Similarly, Boore et al. (2015) also suggested that some *Salmonella* serotypes, including Javiana, that are more commonly isolated from younger patients and in defined geographical locations, may be attributed to natural reservoirs such as reptiles or amphibians [3]. Other investigators have also reported that reptile-associated serotypes have been shown to disproportionately affect young boys [22,23]. This assertion was consistent with our findings, since nearly 33% of all the study isolates originated from male subjects under the age of 10 versus 21% from female subjects in the same age group (Figure 2). Food may also be a reservoir for illnesses; in Germany, *S*. Javiana was one of three major serovars involved in outbreaks associated with contaminated paprika powder and it was the most prevalent infection in children below 14 years [24]. 

There are some important caveats to consider in examining demographic information related to reported foodborne infections. An important feature to consider is that the numbers of reported cases may be more heavily weighted to the pediatric patients due to medical care patterns. It was estimated by the CDC that the under-reporting of cases of salmonellosis in the general population is twice that of younger children [25]. For example, for each laboratory-confirmed case of *Salmonella* infection in children less than five, there are 12 that go unreported, compared to 29 unreported cases for the general population [25]. The exact impact of under-reporting on the current study is not known; however, it could potentially have contributed to the higher prevalence of *S.* Javiana infections identified among children. 

Molecular subtyping was used to determine the distribution of common genotypes and examine the overall genetic diversity between the isolates retrieved from different geographic locations. A total of nine major PFGE clusters with identical *XbaI* restriction profiles were identified (Supplemental Figure S1) and analyzed following *AvrII* restriction. The spatial and temporal distribution of *S.* Javiana types (sharing identical *XbaI* and *AvrII* profiles) in human populations for each cluster is highlighted in Figure 3. The bacterial types were mostly distributed in the CEN, NE and NW regions of Arkansas (Figure 1 and Figure 3). Clusters IV, VI and VII exhibited the widest distribution of clonality among the isolates. Based on the year of isolation, the data suggested that multiple types of *S*. Javiana seem to persist in human populations for several years. For example, among the 22 clonal isolates (labelled in red in Figure 3 and Supplemental Figure S1) in Cluster III, isolates were collected each year from 2003 to 2008. Likewise, the 18 red-labelled *S.* Javiana types in Cluster IV were isolated from 2003 to 2007 in Arkansas and Missouri. Selected types of *S*. Javiana related to these isolates in Arkansas were also observed in Alabama (Cluster I), Oklahoma (Cluster III), and Missouri (Cluster IV). The geographical location of some types from Clusters II, IV, VI, VII and IX could not be identified because of the unavailability of epidemiological data (Figure 3).

Recent studies have also used PFGE profile similarity to link cases to common sources. In an outbreak among workers in a children’s hospital, isolates from 100 of 101 culture-confirmed cases of *S.* Javiana infections had identical PFGE patterns [18]. Another study demonstrated PFGE profile similarity between environmental isolates and clinical isolates, suggesting a linkage between clinical cases and contaminated tomatoes in Virginia [26]. Their findings indicated a long-term reservoir for persistent and endemic contamination of this environment. Our PFGE data also showed distinct types of *S*. Javiana appeared to persist in human populations for multiple years. For example, cluster IV of the *XbaI* PFGE analysis includes 47 isolates from all different locations in Arkansas (NE, NW, CEN, SE, and SW) (Figure 3). Mezal et al. (2013) examined *S.* Javiana isolates collected from Arkansas in the years directly following those of the current study (2009–2011) and they also showed the presence of isolates from human patients in 2011 that had *Xba*I PFGE profiles that appeared visually indistinguishable to those in cluster IV [27]. The persistence of strains with identical PFGE patterns over several years may indicate the presence of dominant genotypes and/or reservoirs that may have contributed to the rise in the number of human illnesses reported during the study period. 

While there were dominant types, our PFGE data in general showed no direct correlation between each cluster and the epidemiology of the bacterial isolates, suggesting that the bacterial populations were genetically dispersed. This finding is supported by Mezal et al. (2013), who identified 34 *Xba*I PFGE patterns among different sources, including human clinical samples grouped into five clusters [27].

Multidrug-resistant *Salmonella* can create a major problem for the treatment of illnesses. In this study, only 8% of isolates were susceptible to all tested antimicrobials; the others were resistant to at least one of the antimicrobials tested with the highest percent resistance to SUL (89%). Additional isolates (n = 11) showed resistance to at least one antimicrobial including GEN, STR, CHL, TET, NAL, CIP, or TIO (third generation cephalosporins) alone or co-resistant with SUL (Table 3). The current study’s findings of extensive SUL resistance, that may also include resistance to TET or FOX, was also observed among *S*. Javiana isolated from irrigation water and soil on a tomato farm [28]. In another study, *S.* Javiana isolates from food and human clinical sources showed intermediate resistance to GEN and STR, while some clinical isolates were also resistant to TET [27]. 

The PCR-based replicon typing assay detected four plasmid Inc types (p-1α, T, W or I1). In a previous study, four clinical and two food-associated *S.* Javiana isolates carried one or more plasmids of approximately 30, 38, and 58 kb, with incompatibility group IncFIIA; while other clinical *S.* Javiana isolates were shown to carry IncI1-type plasmids [27]. In our study, four isolates possessed >90 kb plasmids that were likely IncI1, three showed resistance to SUL, and all but one was resistant to STR or TET (Figure 4). IncI1-resistant plasmids have previously been shown to encode resistance to antimicrobials including GEN, TET and STR [29,30]. 

Since the number of infections caused by *S.* Javiana in the U.S. increased significantly over the years of study, it was important to characterize the virulence genes in the isolates that may contribute to illnesses in humans. In this study, PCR analysis of virulence genes indicated that *S*. Javiana harbored several genes, including *hilA, fimH, invA, aceK, spiA, pagC, cdtB, msgA, sipB, prgH, tolC, spaN, orgA*, and *iroN,* that are typically present in virulent *Salmonella* strains (Figure 5). None of the tested isolates were positive for *spvB* and *lpfC* genes. In another study, 50 human clinical and environmental *S*. Javiana isolates harbored multiple virulence genes, including *spiA, pagC, msgA, invA, sipB, prgH, spaN, orgA, tolC, iroN,* and *cdtB;* however, all isolates were negative for *spvB* and *lpfC* genes [31]. The cytolethal distending toxin B, encoded by *cdtB*, is a well-characterized virulence factor among serovar Typhi strains, however, is not as commonly detected among the non-typhoidal serovars, with a few notable exceptions including *S.* Javiana [32]. The potential contribution of *cdtB* in *S.* Javiana pathogenicity is associated with increased cytotoxicity compared to strains lacking the gene [32]. Overall, it appears that *S.* Javiana strains often possess several virulence factors that can contribute to the development of serious infections in humans. Recent studies have shown that more than 40 *S*. Javiana strains possessed CDT genes [33]. Although very little is known about the role of CDT in nontyphoidal *Salmonella*, Miller et al. (2018) have recently reported that despite the genetic similarity of CDT genes, *S*. Javiana has different genetic conditions than *S*. Typhi for the production and export of functional CDT [33]. 

Furthermore, in this study, the WGS showed that most of our isolates possessed 8 SPIs genes, including SPI-1, SPI-2, SPI-3, SPI-4, SPI-5, SPI-9, SPI-13, and SPI-14, as well as C63PI, out of 23 known SPIs [34]. The SPI-1 and the SPI-2 play a potential role in intestinal invasion and development of enteritis, since they encode type III secretion systems (T3SS) [35,36]. SPI-1 or SPI-2 genes also co-regulate the SPI-5 genes and encode the effector proteins for the T3SS [37]. Genes encoded on SPI-3 are vital for gut colonization and intracellular survival [38], genes encoded on SPI-4 and SPI-9 are necessary for epithelial cell adhesion [39], and genes encoded on SPI-13 are important for intracellular viability [40]. Further, SPI-14 play a role in the activation of SPI-1 genes and mediates bacterial invasion [41].

To understand the evolutionary relatedness of *S*. Javiana in the current study, whole genome sequence data was used to develop a phylogenetic-based evolutionary tree, showing that although the eighteen sequenced isolates (Table 4) shared similar virulence factor profiles, five carried very identical virulence genes, including *rfaI,* meaning that all descendants of isolates shared the last common ancestor (LCA) (Figure 6; red box). To assess the evolutionary and temporal context of *S*. Javiana isolates, it was necessary to compare the isolates originating from Arkansas from 2003 to 2011, with the isolates that collected from different areas in the U.S. in 1999. We found that these isolates, collected in 1999, were grouped in a phylogenetic clade with those collected between 2003 to 2011. These strains shared the LCA, and they carried similar virulence and antibiotic resistance genes (Table 5). Similarly, in the previous report, SNP analysis of genomes of *S*. Typhimurium strains clustered together based on genetic relatedness [42]. Overlapping genotypes of the isolates of the current study with those isolates in 1999 indicated that *S*. Javiana strains have persisted in the environment, which potentially leads to the dissemination of virulence and antimicrobial resistance genes.

SNP analysis revealed the genetic structure of *S*. Javiana isolates clustered based on the geographic origin of Arkansas (Figure 6, blue, green, and purple box). Similarly, in the previous study, *S*. Typhimurium isolates clustered in three monophyletic groups based on host origin [42].

In conclusion, *Salmonella* Javiana has emerged as one of the top serotypes associated with human illnesses in the early part of this century [43]. This study demonstrated that strains with apparently identical genotypes were associated with human illnesses over multiple years during a time when the incidence rate of infections nearly doubled. Most of the isolates studied remained susceptible to most clinically relevant antimicrobials, with less than 2% displaying resistance to more than a single antimicrobial tested. Furthermore, both antimicrobial-resistant and susceptible isolates harbored multiple virulence genes that may contribute to the disease-causing potential of this *Salmonella* serotype in food and human outbreak scenarios. All the isolates analyzed for the presence of potential virulence factors were positive for *cdtB*, which has been shown in other studies to increase the cytotoxicity of strains in in vitro studies [32]. Deeper investigations into the epidemiology of *S.* Javiana infections and the specific genetics of strains that may have facilitated its emergence as a prominent human pathogen may help to better our understanding of *Salmonella* to develop more targeted prevention efforts to limit infections.

## 4. Materials and Methods

### 4.1. Bacterial Strains 

This retrospective study was conducted on four hundred nine *Salmonella enterica* serovar Javiana strains isolated from human patients during the years 2003 to 2008, which were obtained from the Arkansas Department of Health in Little Rock, AR to better understand the microbial genetics and clonal dissemination of *S.* Javiana strains over years. The isolates were stored in brain heart infusion broth with 25% glycerol at −70 °C. The isolates were streaked on tryptic soy agar with 5% sheep blood (Remel, Lenexa, KS) and incubated at 37 °C for 18 to 24 h before analysis.

### 4.2. Pulsed-Field Gel Electrophoresis (PFGE)

The PFGE analysis was performed on a CHEF-Mapper XA system (Bio-Rad Laboratories, Hercules, CA, USA) using the CDC’s PulseNet protocol [44]. The enzymes *Xba*I and *AvrII* (*Bln*I) were used for digestion and analysis. Isolates with identical fingerprint profiles with both enzymes were termed “types”. The restriction digestion patterns were analyzed using BioNumerics software version 4.61 (Applied Maths, Sint-Martens-Latem, Belgium) as previously reported [31]. The similarity matrix and clustering were calculated using the Dice coefficient and the unweighted pair group method using arithmetic means (UPGMA) algorithm, respectively. A positional tolerance shift of 1.5% was allowed between similar bands, and an optimization shift of 1.5% was allowed between any two patterns while generating the dendrograms [31].

### 4.3. Antimicrobial Susceptibility Testing (AST)

The bacterial isolates were tested for AST by broth micro-dilution following Clinical and Laboratory Standards Institute (CLSI) guidelines [45,46] on CMV1AGNF plates with the Sensititre system (Trek Diagnostics, Cleveland, OH, USA). The panel assayed for susceptibility to the following 15 antimicrobials: amikacin (AMI), amoxicillin-clavulanic acid (AMC), ampicillin (AMP), cefoxitin (FOX), TIO, ceftriaxone (AXO), CIP, CHL, GEN, kanamycin (KAN), NAL, STR, SUL, trimethoprim-sulfamethoxazole (SXT), and TET. *Escherichia coli* ATCC 25,922 and *Staphylococcus aureus* ATCC 29,213 were used as quality control strains. 

### 4.4. Plasmid Analysis and Replicon Typing

Plasmid DNA was isolated from a subset of strains that had varying AST and/or PFGE profiles using the Qiagen Miniprep kit (Qiagen Inc., Valencia, CA, USA) following the manufacturer’s instructions. The plasmid DNA was separated on 0.7% LE agarose gels (Lonza, Rockland, ME, USA) in 1X TBE buffer (Bio-Rad) and stained with GelStar nucleic acid stain (Lonza). The plasmid sizes were determined by comparing with BAC-Tracker ladder (8 to 165 kb range: Epicentre, Madison, WI, USA) and exACTGene 1 kb DNA ladder (Fisher Scientific, Pittsburgh, PA, USA) for smaller plasmids. 

Plasmids were also characterized using a PCR-based replicon typing method, which was used to predict the plasmid incompatibility (Inc) groups using a previously published protocol [47], with positive controls provided by Alessandra Carattolli [48]. An aliquot (10 μL) of the amplified PCR product was loaded into a well of a 2% E-gel 48 with ethidium bromide (Invitrogen) and electrophoresed for 20 to 30 min along with the exACTGene 100-bp DNA ladder (Fisher Scientific) for size determination.

### 4.5. Detection of Virulence Genes

PCR was used to determine the presence of multiple virulence genes using previously reported primer sets, including: *16urr, rmbA, spi4H, ttrB* [49], *iss* [50], *fimH, hilA, pefB* [51], *virB4, virD4* [52], *aceK, h-li, invA, sopB* [53], *iucC, iutA, sitA* [54], *cdtB, iroN, lpfC, msgA, orgA, pagC, prgH, sifA, sipB, 16urr, spiA, spvB, tolC* [55], *avrA, iacP, rhuM, sopE,* and *sugR* [56]. Genomic DNA was isolated using the Wizard Genomic DNA Purification Kit according to the manufacturer’s instructions (Promega Corp., Madison, WI, USA). The DNA concentration was determined using the Nanodrop ND-1000 spectrophotometer (Nanodrop Technologies, Wilmington, DE). Amplification was performed in a 25 µL reaction that included 12.5 µL 2X PCR Master Mix (Promega), 1 µL of each of 0.1 mM forward and reverse primers (Eurofins MWG, Huntsville, AL, USA), 1 µL of template DNA, and 9.5 µL nuclease-free water. Reactions were performed in a GeneAmp PCR System 9700 (Applied Biosystems) using the following conditions: 5 min at 95 °C, 34 cycles of 30 s at 94 °C, 30 s at X °C, and 2 min at 72 °C, with a final cycle of 10 min at 72 °C, where X = 66.5 °C for *spvB, spiA, pagC, cdtB, msgA*, *sipB, prgH, 16urr, orgA, tolC, iroN, lpfC*, and *sifA*; 50 °C for *iss*, *fimH, invA*, and *h-li*; 54 °C for *rmbA, avrA, rhuM, ttrB, spi*4H*, iacP, sopE, 16urr, and sugR*; and 58 °C for *iucC, iutA, hilA, virD*4, *sitA, sopB, virB*4, *pefB*, and *aceK* (Table S1). Each PCR product was loaded onto a 2% E-gel and electrophoresed using molecular weight standards and positive controls.

### 4.6. Whole Genome Sequencing (WGS)

The WGS of selected *Salmonella* Javiana isolates (n = 18) was performed following the protocol previously described [42]. Genomic DNA was extracted using a Dneasy Blood and Tissue kit (Qiagen, Valencia, CA, USA). DNA quality and quantity were measured using a Nanodrop (ThermoFisher Scientific, Grand Island, NY, USA) and Qubit BR assay (ThermoFisher Scientific). DNA libraries were constructed using 1 ng DNA from each sample using Nextera XT DNA library preparation kits (Illumina, San Diego, CA, USA). Samples were multiplexed using combinations of two indexes of Nextera XT Index Kit (Illumina). DNA samples were diluted, denatured, and loaded on an Illumina MiSeq instrument with a 2 × 250 pair-end format. Samples were sequenced in multiple batches.

### 4.7. Bioinformatics and Phylogenetic Analyses

Genome sequences from 18 *S*. Javiana isolates were trimmed, and de novo assembly was completed using CLC Genomics Workbench (version. 9.0, Qiagen, Redwood City, CA, USA). FASTA files of sequence assemblies from each strain were analyzed using PlasmidFinder (version 2.1) and ResFinder (version 4.1) to identify predicted plasmids and antimicrobial resistance genes, respectively [57,58]. Pathosystems Resource Integration Center (PATRIC-database) was used to identify distribution of virulence genes and phylogenetic analyses. [59].

## Figures and Tables

**Table 1:** Distribution of *S*. Javiana isolates based on zones and U.S. states.

STATE	Isolates	Percentage	AR REGION	Isolates	Percentage
**Arkansas**	394	96.3	**Central**	168	42.6
**Alabama**	1	0.2	**Northeast**	134	34.0
**California**	2	0.5	**Northwest**	57	14.5
**Missouri**	2	0.5	**Southeast**	14	3.6
**Oklahoma**	4	1.0	**Southwest**	13	3.3
**Texas**	1	0.2	**Unknown**	8	2.0
**Unknown**	5	1.2	**Total**	**394**	**100**
**Total**	**409**	**100**			

**Table 2:** Distribution of the *S*. Javiana isolates based on sex, age and year of isolation.

SEX	Isolates	Percentage	AGE	Isolates	Percentage	YEAR	Isolates	Percentage
**Female**	177	43.3	**<1 to 10**	221	54.0	**2003**	101	24.7
**Male**	231	56.5	**11 to 20**	29	7.1	**2004**	96	23.5
**Unknown**	1	0.2	**21 to 30**	15	3.7	**2005**	42	10.3
**Total**	**409**	**100**	**31 to 40**	20	4.9	**2006**	49	12.0
			**41 to 50**	18	4.4	**2007**	71	17.4
			**51 to 60**	28	6.8	**2008**	50	12.2
			**61 to 70**	22	5.4	**Total**	**409**	**100**
			**71 to 80**	23	5.6			
			**81 to 90**	6	1.5			
			**Unknown**	27	6.6			
			**Total**	**409**	**100**			

**Table 3:** Antimicrobial resistance profiles of *S.* Javiana isolates from clinical cases in Arkansas in human populations.

Antimicrobial Resistance Profile	Number of Isolates	% Prevalence
Pan-susceptible	34	8
SUL only	364	89
SUL, STR, GEN	1	<1
SUL, TET, GEN	1	<1
SUL, CHL	1	<1
SUL, STR	2	<1
SUL, TIO	1	<1
NAL, CIP	1	<1
TET	1	<1
STR	2	<1
NAL	1	<1
**TOTAL**	**409**	**100**

**Table 4:** SPI, virulence, and antimicrobial resistance genes based on WGS analysis.

Isolates	Location	Virulence Genes	SPI Genes	Resistance Genes	Phenotype
JV-333	AR, CEN	*sopAD2, fimYD, pagOD, sitA, iroB, pipBACD, sspA, rfaJ*	*SPI-2, SPI-13, SPI-14, C63PI*	aac(6’)-Iaa	Aminoglycoside resistance
JV-334	AR, NE	*sopAD2, fimYD, pagO, sitA, iroB, pipBACD, sspA, rfaJI*	*SPI-2, SPI-13, SPI-14, C63PI*	aac(6’)-Iaa	Aminoglycoside resistance
				parC p.T57S	Nalidixic acid, Ciprofloxacin
JV410	MI	*sopAD2, fimYD, pagOD, sitA, iroB, pipBACD, sspA, rfaJ*	*SPI-1, SPI-2, SPI-3, SPI-4, SPI-5, SPI-9, SPI-13, SPI-14, C63PI*	aac(6’)-Iaa	Aminoglycoside resistance
				parC p.T57S	Nalidixic acid, Ciprofloxacin
JV421	AR, NE	*sopAD2, fimYD, pagOD, sitA, iroB, pipBACD, sspA, rfaJI*	*SPI-1, SPI-2, SPI-3, SPI-4, SPI-5, SPI-9, SPI-13, SPI-14, C63PI*	aac(6’)-Iaa	Aminoglycoside resistance
				parC p.T57S	Nalidixic acid, Ciprofloxacin
JV426	AR, CEN	*sopAD2, fimYD, pagOD, sitA, iroB, pipBACD, sspA, rfaJ*	*SPI-1, SPI-2, SPI-3, SPI-4, SPI-5, SPI-9, SPI-13, SPI-14, C63PI*	aac(6’)-Iaa	Aminoglycoside resistance
				parC p.T57S	Nalidixic acid, Ciprofloxacin
JV435	GA	*sopAD2, fimYD, pagOD, sitA, iroB, pipBACD, sspA,* *rfaI*	*SPI-1, SPI-2, SPI-3, SPI-5, SPI-9, SPI-13, SPI-14, C63PI*	aac(6’)-Iaa	Aminoglycoside resistance
				parC p.T57S	Nalidixic acid, Ciprofloxacin
JV475	USA	*sopAD2, fimYD, pagOD, sitA, iroB, pipBACD, sspA, rfaJI*	*SPI-1, SPI-2, SPI-3, SPI-4, SPI-9, SPI-13, SPI-14, C63PI*	aac(6’)-Iaa	Aminoglycoside resistance
				parC p.T57S	Nalidixic acid, Ciprofloxacin
JV497	AR, NE	*sopAD2, fimYD, pagOD, sitA, iroB, pipBACD, sspA*	*SPI-1, SPI-2, SPI-3, SPI-4, SPI-5, SPI-9, SPI-13, SPI-14, C63PI*	aac(6’)-Iaa	Aminoglycoside resistance
				parC p.T57S	Nalidixic acid, Ciprofloxacin
JV529	AR, NE	*sopAD2, fimYD, pagO, sitA, iroB, pipBACD, sspA rfaI,*	*SPI-1, SPI-2, SPI-3, SPI-4, SPI-5, SPI-9, SPI-13, SPI-14, C63PI*	aac(6’)-Iaa	Aminoglycoside resistance
				parC p.T57S	Nalidixic acid, Ciprofloxacin
JV547	AR, CEN	*sopAD2, fimYD, pagOD, sitA, iroB, pipBACD, sspA, rfaJI*	*SPI-1, SPI-2, SPI-3, SPI-4, SPI-5, SPI-9, SPI-13, SPI-14, C63PI*	aac(6’)-Iaa	Aminoglycoside resistance
				parC p.T57S	Nalidixic acid, Ciprofloxacin
JV624	AR, CEN	*sopAD2, fimYD, pagOD, sitA, iroB, pipBACD, sspA, rfaJ*	*SPI-1, SPI-2, SPI-3, SPI-4, SPI-5, SPI-9, SPI-13, SPI-14, C63PI*	aac(6’)-Iaa	Aminoglycoside resistance
				parC p.T57S	Nalidixic acid, Ciprofloxacin
JV652	AR, NE	*sopAD2, fimYD, pagOD, sitA, iroB, pipBACD, sspA, rfaJI*	*SPI-1, SPI-2, SPI-3, SPI-4, SPI-5, SPI-9, SPI-13, SPI-14, C63PI*	aac(6’)-Iaa	Aminoglycoside resistance
				parC p.T57S	Nalidixic acid, Ciprofloxacin
JV699	USA	*sopAD2, fimYD, pagOD, sitA, iroB, pipBACD, sspA, rfaI*	*SPI-1, SPI-2, SPI-3, SPI-4, SPI-5, SPI-9, SPI-13, SPI-14, C63PI*	aac(6’)-Iaa	Aminoglycoside resistance
				parC p.T57S	Nalidixic acid, Ciprofloxacin
JV726	AR, CEN	*sopAD2, fimYD, pagOD, sitA, iroB, pipBACD, sspA, rfaJ*	*SPI-1, SPI-2, SPI-3, SPI-4, SPI-5, SPI-9, SPI-13, SPI-14, C63PI*	aac(6’)-Iaa	Aminoglycoside resistance
				parC p.T57S	Nalidixic acid, Ciprofloxacin
JV841	AR, NE	*sopAD2, fimYD, pagOD, sitA, iroB, pipBACD, sspA, rfaJ*	N/A	aac(6’)-Iaa	Aminoglycoside resistance
				parC p.T57S	Nalidixic acid, Ciprofloxacin
JV878	AR, NE	*sopAD2, fimYD, pagOD, sitA, iroB, pipBACD, sspA, rfaJI*	N/A	aac(6’)-Iaa	Aminoglycoside resistance
				parC p.T57S	Nalidixic acid, Ciprofloxacin
JV910	AR, NE	*sopAD2, fimYD, pagOD, sitA, iroB, pipBACD, sspA, rfaJI*	N/A	aac(6’)-Iaa	Aminoglycoside resistance
				parC p.T57S	Nalidixic acid, Ciprofloxacin
JV1006	AR, NE	*sopAD2, fimYD, pagOD, sitA, iroB, pipBACD, sspA, rfaJI*	N/A	aac(6’)-Iaa	Aminoglycoside resistance
				parC p.T57S	Nalidixic acid, Ciprofloxacin

**Table 5:** Isolates information for 18 *S. enterica* serovar Javiana isolates sequenced.

Isolate	Source	Isolation Location	Year	GenBank Accession
333	Clinical	AR	2005	JAHWXF000000000
334	Clinical	AR	2005	JAHWXE000000000
410	Clinical	MI	1999	JAHWXD000000000
421	Clinical	AR	2004	JAHWXC000000000
426	Clinical	AR	2004	JAHWXB000000000
435	Clinical	GA	1999	JAHWXA000000000
475	Clinical	USA	1999	JAHWWZ000000000
497	Clinical	AR	2004	JAHWWY000000000
529	Clinical	AR	2003	JAHWWX000000000
547	Clinical	AR	2003	JAHWWW000000000
624	Clinical	AR	2006	JAHWWV000000000
652	Clinical	AR	2007	JAHWWU000000000
699	Clinical	USA	1999	JAHWWT000000000
726	Clinical	AR	2008	JAHWWS000000000
841	Clinical	AR	2011	JAHWWR000000000
878	Clinical	AR	2011	JAHWWQ000000000
910	Clinical	AR	2011	JAHWWP000000000
1006	Clinical	AR	2011	JAHWWO000000000

## Supplementary Materials

The following supporting information can be downloaded at: https://www.mdpi.com/article/10.3390/pathogens11101192/s1, Figure S1: Dendrogram based on *XbaI* and *AvrII* pulsed-field gel electrophoresis macro-restriction profiles of *Salmonella* Javiana strains. The dendrogram on the left illustrates *XbaI* digested profiles, while the dendrograms on the right illustrate *AvrII* digested profiles for the corresponding cluster; Table S1: *Salmonella enterica* serovar Javiana virulence gene targets, their function, primer sequences and amplicon sizes used in this study.
