# Supplementary material for: Microbial Genetics and Clonal Dissemination of Salmonella enterica Serotype Javiana Isolated from Human Populations in Arkansas, USA
Source: Pathogens. 2022 Oct 16;11(10):1192. doi: 10.3390/pathogens11101192 (PMC9611979; doi:10.3390/pathogens11101192)
Supplement: Supplementary file 1 [file pathogens-11-01192-s001.zip › pathogens-1937544-supplementary.pdf]

Supplemental Table S1 *Salmonella enterica* serovar Javiana virulence gene targets, their function, primer sequences and amplicon sizes used in this study

Target gene	Function	Oligonucleotide sequence	Amplicon size	Reference
spvB	Growth within host	F: CTATCAGCCCCGCACGGAGAGCAGTTTTTA<br>R: GGAGGAGGCGGTGGCGGTGGCATCATA	717	Skyberg, Av. Dis, 2006
spiA	Survival within macrophage	F: CCAGGGGTCGTTAGTGTATTGCGTGAGATG<br>R: CGCGTAACAAAGAACCCGTAGTGATGGATT	550	Skyberg, Av. Dis, 2006
pagC	Survival within macrophage	F: CGCCTTTTCCGTGGGGTATGC<br>R: GAAGCCGTTTATTTTTGTAGAGGAGATGTT	454	Skyberg, Av. Dis, 2006
cdtB	Host recognition/invasion	F: ACAACTGTCGCATCTCGCCCCGTCATT<br>R: CAATTTGCGTGGGTTCTGTAGGTGCGAGT	268	Skyberg, Av. Dis, 2006
msgA	Survival within macrophage	F: GCCAGGCGCACGCGAAATCATCC<br>R: GCGACCAGCCACATATCAGCCTCTTCAAAC	189	Skyberg, Av. Dis, 2006
sipB	Entry into nonphagocytic cells, killing of macrophages	F: GGACGCCGCCCGGGAAAAACTCTC<br>R: ACACTCCCGTCGCCGCCTTCACAA	875	Skyberg, Av. Dis, 2006
prgH	Host recognition/invasion	F: GCCCGAGCAGCCTGAGAAGTTAGAAA<br>R: TGAAATGAGCGCCCCTTGAGCCAGTC	756	Skyberg, Av. Dis, 2006
spaN	Entry into nonphagocytic cells, killing of macrophages	F: AAAAGCCGTGGAATCCGTTAGTGAAGT<br>R: CAGCGCTGGGGATTACCGTTTTG	504	Skyberg, Av. Dis, 2006
orgA	Host recognition/invasion	F: TTTTTGGCAATGCATCAGGGAACA<br>R: GGCGAAAGCGGGGACGGTATT	255	Skyberg, Av. Dis, 2006
tolC	Host recognition/invasion	F: TACCCAGGCGCAAAAAGAGGCTATC<br>R: CCGCGTTATCCAGGTTGTTGC	161	Skyberg, Av. Dis, 2006
lpfC	Host recognition/invasion	F: GCCCCGCCTGAAGCCTGTGTTGC<br>R: AGGTCGCCGCTGTTTGAGGTTGGATA	641	Skyberg, Av. Dis, 2006
sifA	Filamentous structure formation	F: TTTGCCGAACGCGCCCCCACACG<br>R: GTTGCCTTTTCTTGCGCTTTCCACCCATCT	449	Skyberg, Av. Dis, 2006
sopB	Host recognition/invasion	F:ATGCAAATACAGAGCTTCTATCA<br>R: GGCATAAAGGGACAGCACA	1700	Nayak, IJFM, 2004
pefB	Host recognition/invasion	F: TGATGCTGAACAGAAAAGAT<br>R: ATAATAAACAACCATGTGAG	296	Foley, JCM, 2006
iroN	Iron acquisition	F: ACTGGCACGGCTCGCTGTCGCTCTAT<br>R: CGCTTTACCGCCGTTCTGCCACTGC	1205	Skyberg, Av. Dis, 2006
sitA	Iron acquisition	F:AAGCGTTGGAACCACAATTC<br>R:GTCCTCACCTGCTCGATAGC	245	

hilA	Host recognition/invasion	F:TTAATCGTCCGGTCGTAGTG<br>R:TCTGCCAGCGCACAGTAAGG	597	Foley, JCM, 2006
fimH	Adhesion to cells	F:GGGACGGCGACCGATATCTT<br>R:TTGTCTGGCGAGGGATCGTC	412	Foley, JCM, 2006
iss	Survival	F:TCACATAGGATTCTGCCG<br>R:AGAAATCAAAAGGTGGCC	607	Dezfulian,JCM, 2003
iutA	Aerobaction receptor	F:GGCTGGACATCATGGGAACTGG<br>R:CGTCGGGAACGGGTAGAATCG	302	
iucC	Iron uptake; intracellular growth	F:GTCGCCGTGGTGGGGTAAGAG<br>R:TCCCCCGGTAGCATGAGGTGTTGT	429	
virB4	Type 4 secretion system	F:TTGGCATTGATGGCACCGAGTTTC<br>R:TGTAATCGGCATCAGGGTTAGCCA	315	
virD4	Type 4 secretion system	F:TCGGCCTCTCAGCTTGAGGAAATCT<br>R:TTTGCAAGTTCCGGAGCAACCTTC	370	
invA	Host recognition/invasion	F:TATCGCCACGTTCGGGCAA<br>R:TCGCACCGTCAAAGGAACC	275	Nayak, IJFM, 2004
h-li	Flagellin	F:AGCCTCGGCTACTGGTCTTG<br>R:CCGCAGCAAGAGTCACCTCA	173	Nayak, IJFM, 2004
aceK	Regulation of isocitrate in tricarboxylic and glyoxylate bypass	F:CAGGGCGAGGGTATGAAACAC<br>R:AACTGCGATTCTTCGGTAGAAC	191	Nayak, IJFM, 2004
iacP	Invasion associated acyl carrier	F:CACCTCTTGTATTGCCGTTG<br>R: GGCATATATCCGCAAAGGTC	176	Zou, unpub. data
sopE	Encodes a effector protein	F: ATTGTTGTGGCGTTGGCATCGT<br>R: AATGCGAGTAAAGATCCGGCCT	186	Zou, unpub. Data
rhuM	Encodes a cytoplasmic protein	F: CATCGGCTGTACCCGACTAT<br>R: CAGCACGCTGATGAATGAGT	222	Zou, unpub. Data
*spi*4H	Inhibits serine peptidase	F: ACTGCTCGCTTGTGGTATCAGGAA<br>R: TCTTACCGTGCTGTGGATGGTTCA	154	Courtney, Mol Cell Probes, 2006
ttrB	Tetrathionate respiration	F: ATGTGGACGGGAGTCAATATGG<br>R: GTGGCGATGCGGCTATGG	608	Courtney, Mol Cell Probes, 2006
sugR	Encodes ATP binding protein	F: ACTGCTCGCTTGTGGTATCAGGAA<br>R: TCTTACCGTGCTGTGGATGGTTCA	152	Zou, unpub. Data

purR	DNA-binding transcriptional repressor	F: CGCTTCCCCTTTTCCTCAAG<br>R: CCATCACCACCATCGGAATATG	629	Courtney, Mol Cell Probes, 2006
rmbA	Encodes a cytoplasmic protein	F: AGCCTTCACAAATTGTCCATTG<br>R: TCCGTATAGTTAAGCGTTCGTC	454	Courtney, Mol Cell Probes, 2006
avrA	Encodes a secreted effector protein	F:AATGGAAGGCGTTGAATCTG<br>R: GAGCTGCTTTGGTCCTCAAC	170	Zou, unpub. Data

Supplemental Figure S1 Dendrogram based on *XbaI* and *AvrII* pulsed-field gel electrophoresis macro-restriction profiles of *Salmonella* Javiana strains. The dendrogram on the left illustrates *XbaI* digested profiles, while the dendrograms on the right illustrate *AvrII* digested profiles for the corresponding cluster

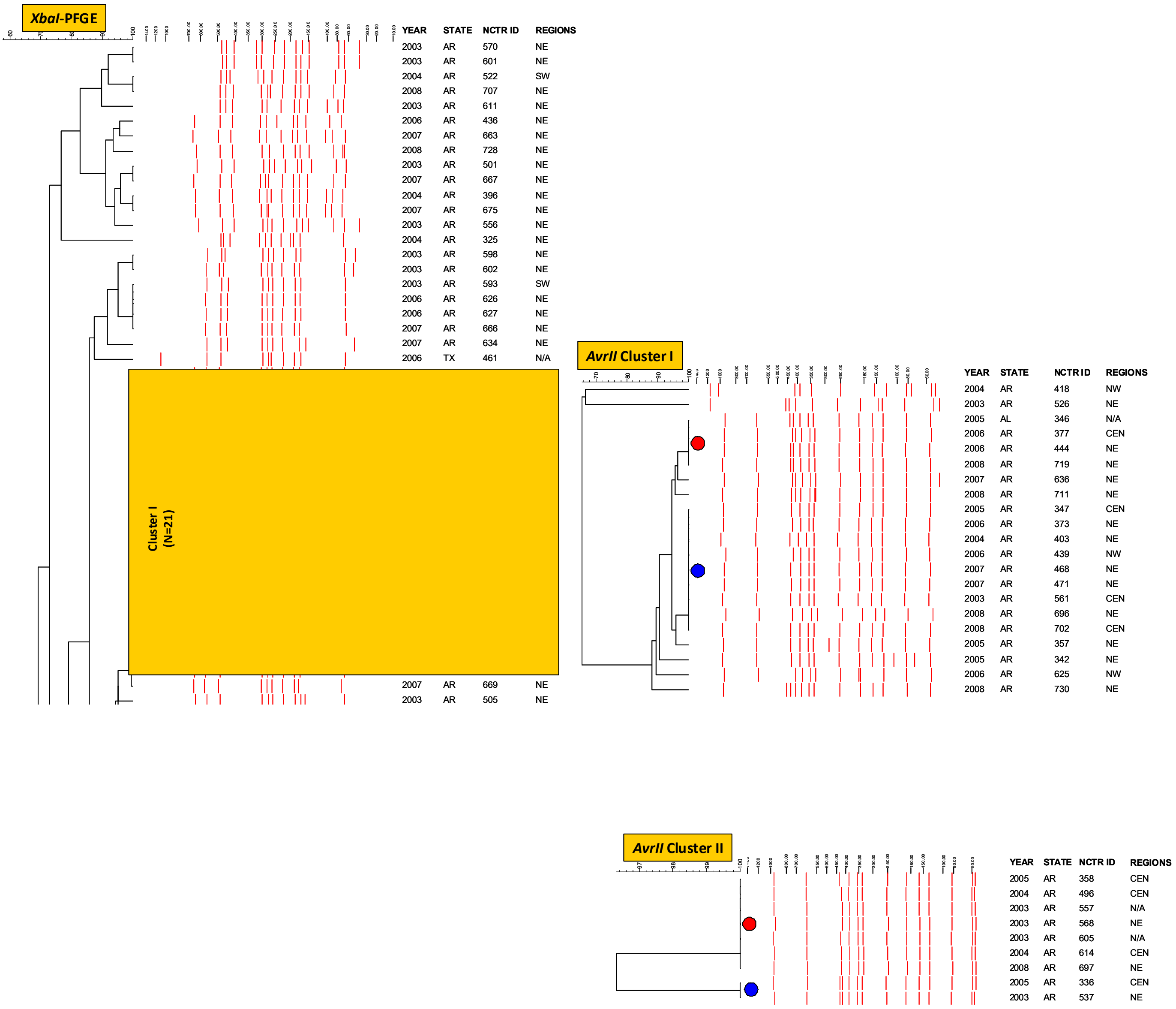

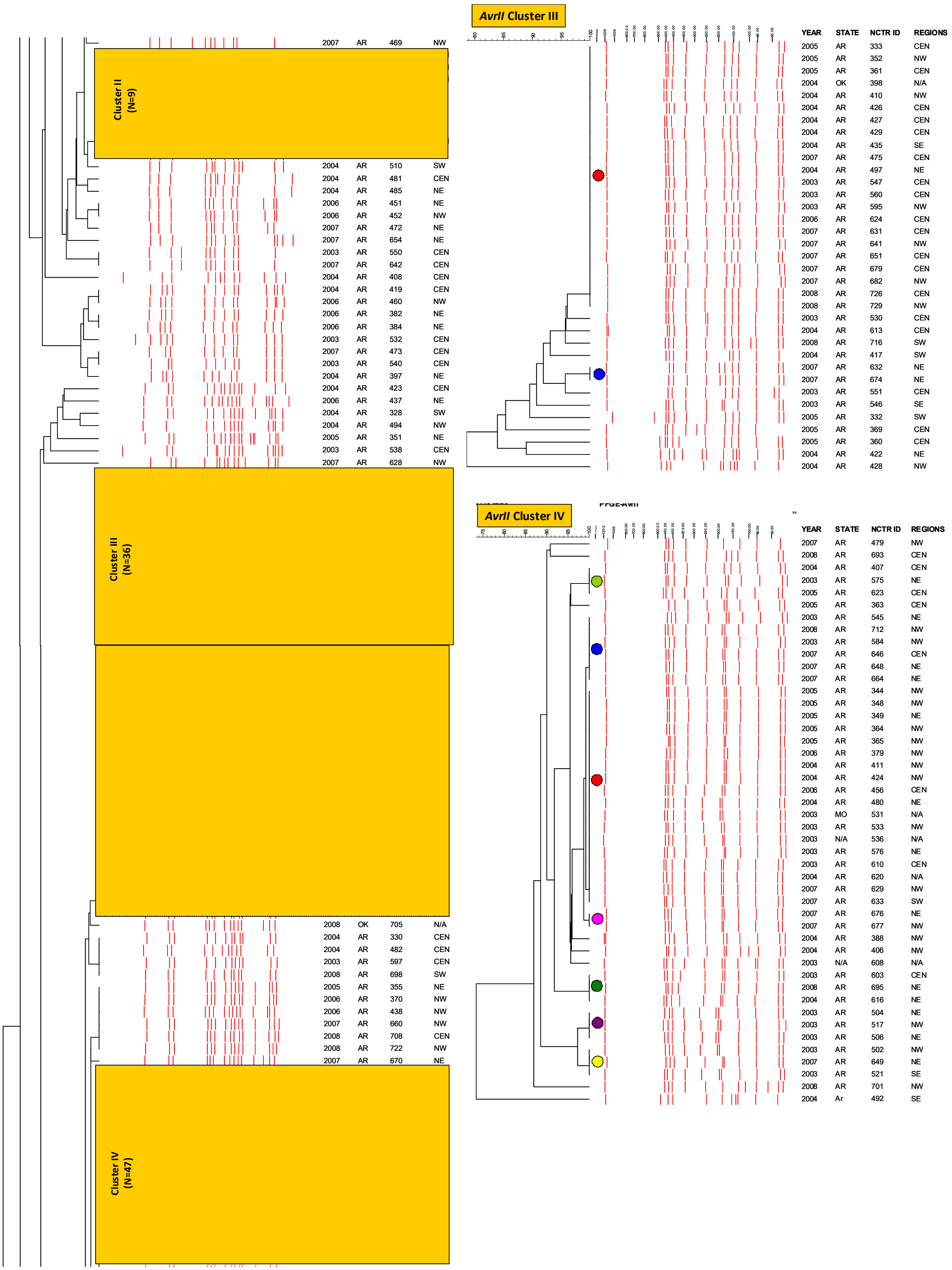
Cluster II
(N=9)
Cluster III
(N=36)
Cluster IV
(N=47)
2007 AR 469 NW
2004 AR 510 SW
2004 AR 481 CEN
2004 AR 485 NE
2006 AR 451 NE
2006 AR 452 NW
2007 AR 472 NE
2007 AR 654 NE
2003 AR 550 CEN
2007 AR 642 CEN
2004 AR 408 CEN
2004 AR 419 CEN
2006 AR 460 NW
2006 AR 382 NE
2006 AR 384 NE
2003 AR 532 CEN
2007 AR 473 CEN
2003 AR 540 CEN
2004 AR 397 NE
2004 AR 423 CEN
2006 AR 437 NE
2004 AR 328 SW
2004 AR 494 NW
2005 AR 351 NE
2003 AR 538 CEN
2007 AR 628 NW
2008 OK 705 N/A
2004 AR 330 CEN
2004 AR 482 CEN
2003 AR 597 CEN
2008 AR 698 SW
2005 AR 355 NE
2006 AR 370 NW
2006 AR 438 NW
2007 AR 660 NW
2008 AR 708 CEN
2008 AR 722 NW
2007 AR 670 NE
AvrII Cluster III
YEAR STATE NCTR ID REGIONS
2005 AR 333 CEN
2005 AR 352 NW
2005 AR 361 CEN
2004 OK 398 N/A
2004 AR 410 NW
2004 AR 426 CEN
2004 AR 427 CEN
2004 AR 429 CEN
2004 AR 435 SE
2007 AR 475 CEN
2004 AR 497 NE
2003 AR 547 CEN
2003 AR 560 CEN
2003 AR 595 NW
2006 AR 624 CEN
2007 AR 631 CEN
2007 AR 641 NW
2007 AR 651 CEN
2007 AR 679 CEN
2007 AR 682 NW
2008 AR 726 CEN
2008 AR 729 NW
2003 AR 530 CEN
2004 AR 613 CEN
2008 AR 716 SW
2004 AR 417 SW
2007 AR 632 NE
2007 AR 674 NE
2003 AR 551 CEN
2003 AR 546 SE
2005 AR 332 SW
2005 AR 369 CEN
2005 AR 360 CEN
2004 AR 422 NE
2004 AR 428 NW
AvrII Cluster IV
YEAR STATE NCTR ID REGIONS
2007 AR 479 NW
2008 AR 693 CEN
2004 AR 407 CEN
2003 AR 575 NE
2005 AR 623 CEN
2005 AR 363 CEN
2003 AR 545 NE
2008 AR 712 NW
2003 AR 584 NW
2007 AR 646 CEN
2007 AR 648 NE
2007 AR 664 NE
2005 AR 344 NW
2005 AR 348 NW
2005 AR 349 NE
2005 AR 364 NW
2005 AR 365 NW
2006 AR 379 NW
2004 AR 411 NW
2004 AR 424 NW
2006 AR 456 CEN
2004 AR 480 NE
2003 MO 531 N/A
2003 AR 533 NW
2003 N/A 536 N/A
2003 AR 576 NE
2003 AR 610 CEN
2004 AR 620 N/A
2007 AR 629 NW
2007 AR 633 SW
2007 AR 676 NE
2007 AR 677 NW
2004 AR 388 NW
2004 AR 406 NW
2003 N/A 608 N/A
2003 AR 603 CEN
2008 AR 695 NE
2004 AR 616 NE
2003 AR 504 NE
2003 AR 517 NW
2003 AR 506 NE
2003 AR 502 NW
2007 AR 649 NE
2003 AR 521 SE
2008 AR 701 NW
2004 Ar 492 SE

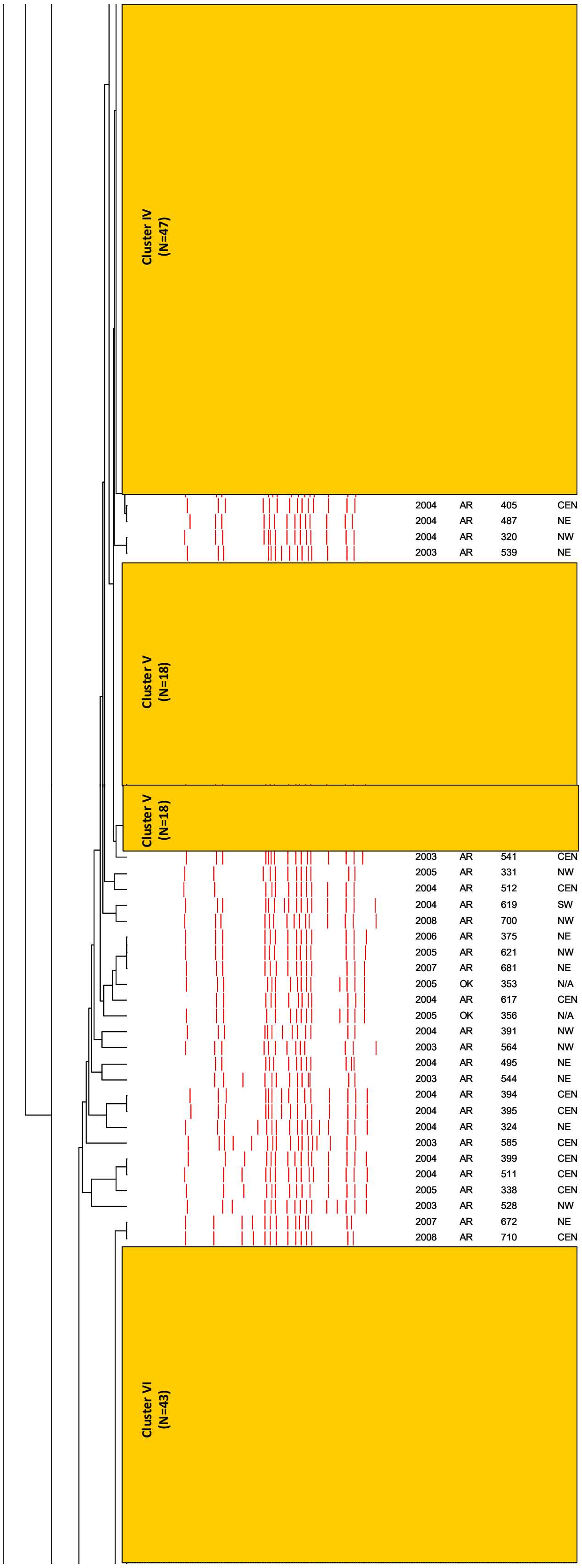

2004	AR	405	CEN
2004	AR	487	NE
2004	AR	320	NW
2003	AR	539	NE
2003	AR	541	CEN
2005	AR	331	NW
2004	AR	512	CEN
2004	AR	619	SW
2008	AR	700	NW
2006	AR	375	NE
2005	AR	621	NW
2007	AR	681	NE
2005	OK	353	N/A
2004	AR	617	CEN
2005	OK	356	N/A
2004	AR	391	NW
2003	AR	564	NW
2004	AR	495	NE
2003	AR	544	NE
2004	AR	394	CEN
2004	AR	395	CEN
2004	AR	324	NE
2003	AR	585	CEN
2004	AR	399	CEN
2004	AR	511	CEN
2005	AR	338	CEN
2003	AR	528	NW
2007	AR	672	NE
2008	AR	710	CEN

***AvrII* Cluster V**

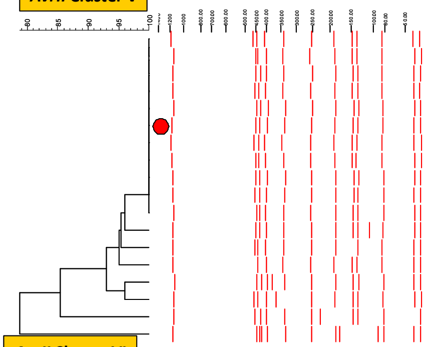

YEAR	STATE	NCTR ID	REGIONS
2004	AR	404	CEN
2004	AR	431	CEN
2006	AR	447	CEN
2006	AR	448	CEN
2004	AR	490	CEN
2003	AR	571	CEN
2003	AR	573	CEN
2007	AR	637	NW
2007	AR	656	NW
2008	AR	714	CEN
2008	AR	732	CEN
2006	AR	446	CEN
2007	AR	650	NE
2003	AR	562	CEN
2008	AR	688	NE
2008	AR	703	CEN
2008	AR	713	N/A
2007	AR	659	CEN

***AvrII* Cluster VI**

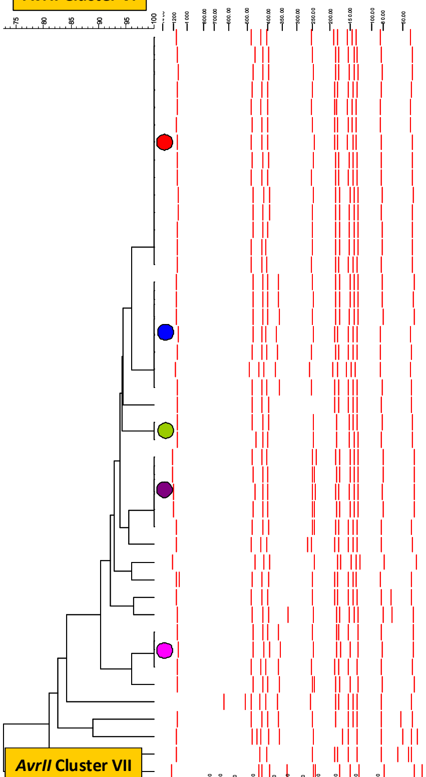

YEAR	STATE	NCTR ID	REGIONS
2006	AR	441	CEN
2006	AR	442	NW
2006	AR	443	NW
2006	AR	445	NW
2006	AR	454	CEN
2007	AR	470	CEN
2003	AR	542	NW
2003	AR	543	NE
2003	AR	549	CEN
2003	AR	554	N/A
2004	AR	615	CEN
2007	AR	655	SE
2007	AR	673	CEN
2008	AR	709	CEN
2005	AR	345	NE
2005	AR	367	CEN
2004	AR	402	NE
2006	AR	449	NE
2006	AR	450	SE
2004	AR	483	NE
2007	AR	658	NE
2006	AR	458	CEN
2004	AR	489	CEN
2008	AR	694	CEN
2004	AR	326	Cen
2005	AR	335	CEN
2005	AR	359	NE
2006	AR	385	NE
2003	AR	518	CEN
2003	AR	574	CEN
2004	AR	430	CEN
2006	AR	457	CEN
2003	AR	572	NE
2007	AR	668	SE
2005	AR	354	CEN
2003	AR	553	SE
2008	AR	715	NE
2005	AR	350	NE
2003	AR	587	NE
2004	AR	488	CEN
2004	AR	493	CEN
2006	AR	453	CEN
2006	AR	440	CEN

***AvrII* Cluster VII**

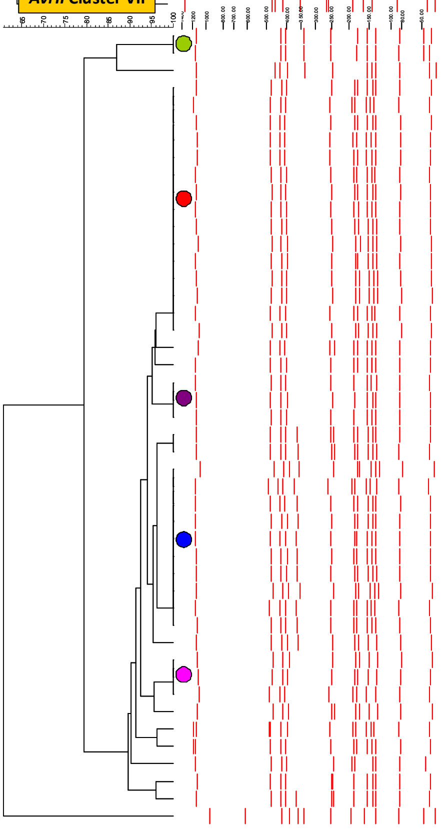

YEAR	STATE	NCTR ID	REGIONS
2004	AR	414	CEN
2003	AR	590	NE
2005	AR	622	NE
2006	AR	378	NE
2004	AR	401	N/A
2004	AR	420	CEN
2004	AR	433	NE
2004	AR	434	CEN
2007	AR	474	CEN
2003	AR	500	CEN
2003	AR	503	CEN
2003	AR	596	NE
2003	AR	604	CEN
2007	AR	643	CEN
2007	AR	665	NE
2008	AR	706	CEN
2008	AR	721	CEN
2008	AR	727	NE
2003	AR	607	CEN
2007	AR	476	CEN
2006	AR	387	CEN
2004	AR	491	CEN
2003	AR	612	CEN
2003	AR	599	NE
2003	AR	600	NE
2005	AR	334	NE
2004	AR	400	NE
2004	AR	421	NE
2007	AR	477	NE
2003	AR	529	NE
2003	AR	569	NE
2003	AR	588	NE
2007	AR	639	NE
2007	AR	652	NE
2008	AR	699	SE
2003	AR	577	NE
2008	AR	684	CEN
2008	AR	689	NE
2008	AR	692	CEN
2008	AR	686	NE
2006	AR	455	CEN
2007	AR	464	CEN
2004	AR	508	CEN
2003	AR	519	SE
2003	AR	524	NE
2003	AR	527	NE

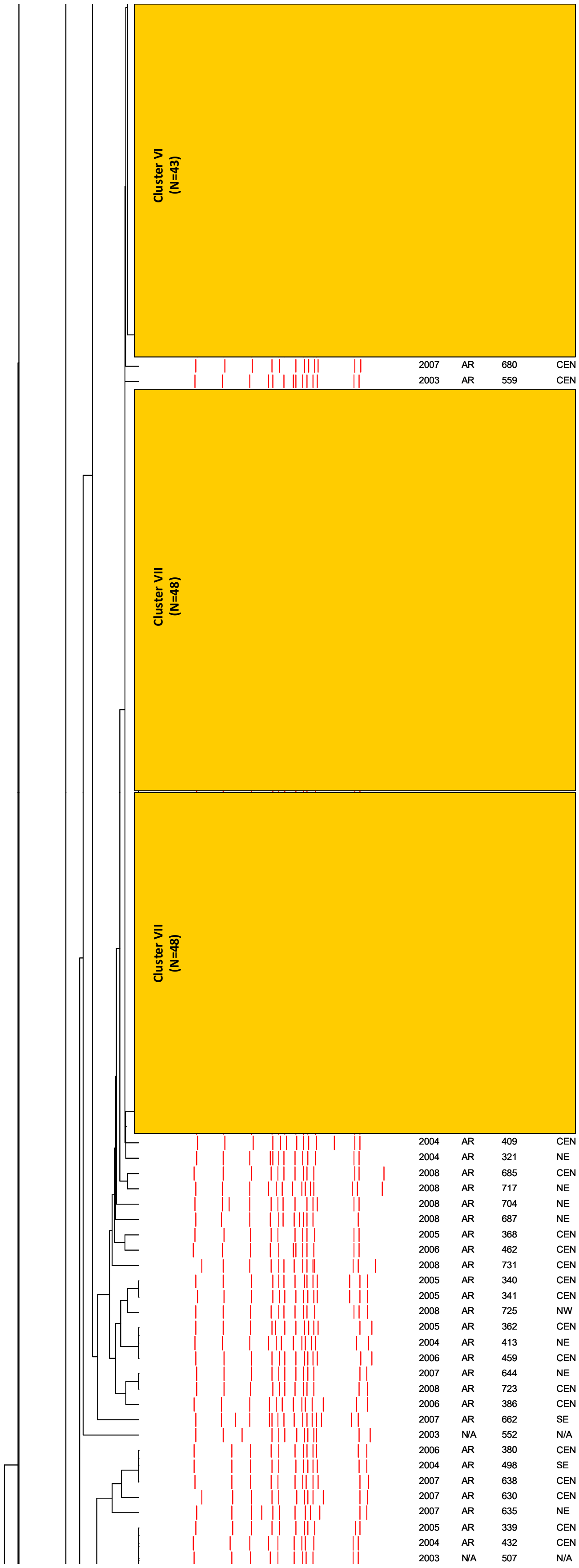
Cluster VI
(N=43)
2007 AR 680 CEN
2003 AR 559 CEN
Cluster VII
(N=48)
Cluster VII
(N=48)
2004 AR 409 CEN
2004 AR 321 NE
2008 AR 685 CEN
2008 AR 717 NE
2008 AR 704 NE
2008 AR 687 NE
2005 AR 368 CEN
2006 AR 462 CEN
2008 AR 731 CEN
2005 AR 340 CEN
2005 AR 341 CEN
2008 AR 725 NW
2005 AR 362 CEN
2004 AR 413 NE
2006 AR 459 CEN
2007 AR 644 NE
2008 AR 723 CEN
2006 AR 386 CEN
2007 AR 662 SE
2003 N/A 552 N/A
2006 AR 380 CEN
2004 AR 498 SE
2007 AR 638 CEN
2007 AR 630 CEN
2007 AR 635 NE
2005 AR 339 CEN
2004 AR 432 CEN
2003 N/A 507 N/A

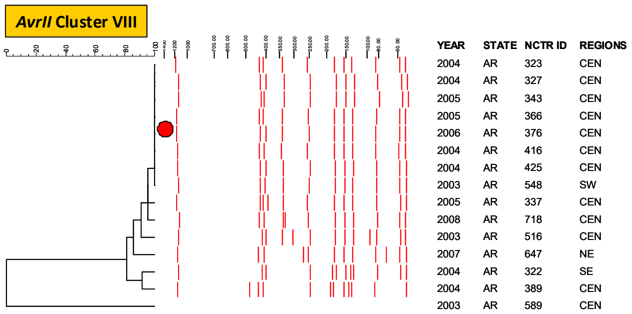
AvrII Cluster VIII
YEAR STATE NCTR ID REGIONS
2004 AR 323 CEN
2004 AR 327 CEN
2005 AR 343 CEN
2005 AR 366 CEN
2006 AR 376 CEN
2004 AR 416 CEN
2004 AR 425 CEN
2003 AR 548 SW
2005 AR 337 CEN
2008 AR 718 CEN
2003 AR 516 CEN
2007 AR 647 NE
2004 AR 322 SE
2004 AR 389 CEN
2003 AR 589 CEN

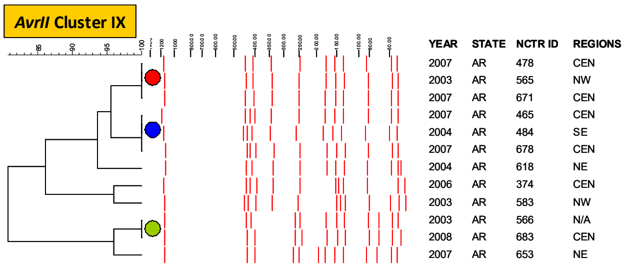
AvrII Cluster IX
YEAR STATE NCTR ID REGIONS
2007 AR 478 CEN
2003 AR 565 NW
2007 AR 671 CEN
2007 AR 465 CEN
2004 AR 484 SE
2007 AR 678 CEN
2004 AR 618 NE
2006 AR 374 CEN
2003 AR 583 NW
2003 AR 566 N/A
2008 AR 683 CEN
2007 AR 653 NE

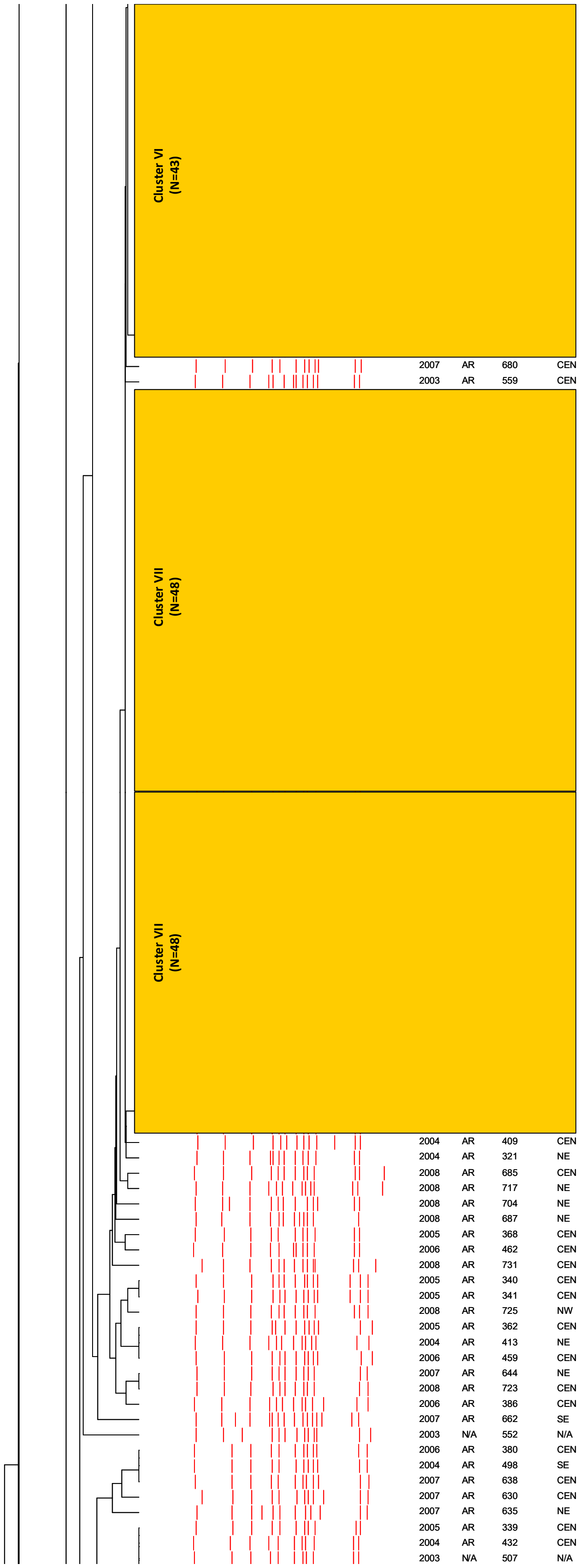

2007	AR	680	CEN
2003	AR	559	CEN
2004	AR	409	CEN
2004	AR	321	NE
2008	AR	685	CEN
2008	AR	717	NE
2008	AR	704	NE
2008	AR	687	NE
2005	AR	368	CEN
2006	AR	462	CEN
2008	AR	731	CEN
2005	AR	340	CEN
2005	AR	341	CEN
2008	AR	725	NW
2005	AR	362	CEN
2004	AR	413	NE
2006	AR	459	CEN
2007	AR	644	NE
2008	AR	723	CEN
2006	AR	386	CEN
2007	AR	662	SE
2003	N/A	552	N/A
2006	AR	380	CEN
2004	AR	498	SE
2007	AR	638	CEN
2007	AR	630	CEN
2007	AR	635	NE
2005	AR	339	CEN
2004	AR	432	CEN
2003	N/A	507	N/A

***AvrII* Cluster VIII**

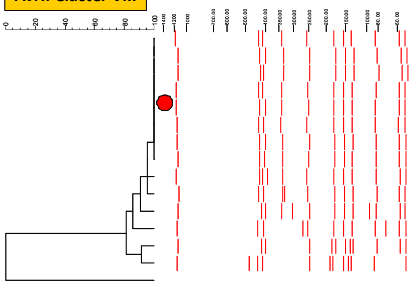

YEAR	STATE	NCTR ID	REGIONS
2004	AR	323	CEN
2004	AR	327	CEN
2005	AR	343	CEN
2005	AR	366	CEN
2006	AR	376	CEN
2004	AR	416	CEN
2004	AR	425	CEN
2003	AR	548	SW
2005	AR	337	CEN
2008	AR	718	CEN
2003	AR	516	CEN
2007	AR	647	NE
2004	AR	322	SE
2004	AR	389	CEN
2003	AR	589	CEN

***AvrII* Cluster IX**

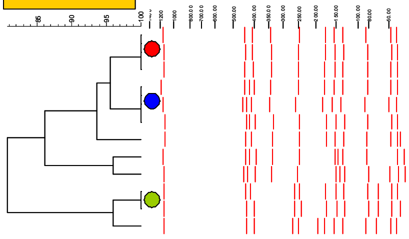

YEAR	STATE	NCTR ID	REGIONS
2007	AR	478	CEN
2003	AR	565	NW
2007	AR	671	CEN
2007	AR	465	CEN
2004	AR	484	SE
2007	AR	678	CEN
2004	AR	618	NE
2006	AR	374	CEN
2003	AR	583	NW
2003	AR	566	N/A
2008	AR	683	CEN
2007	AR	653	NE

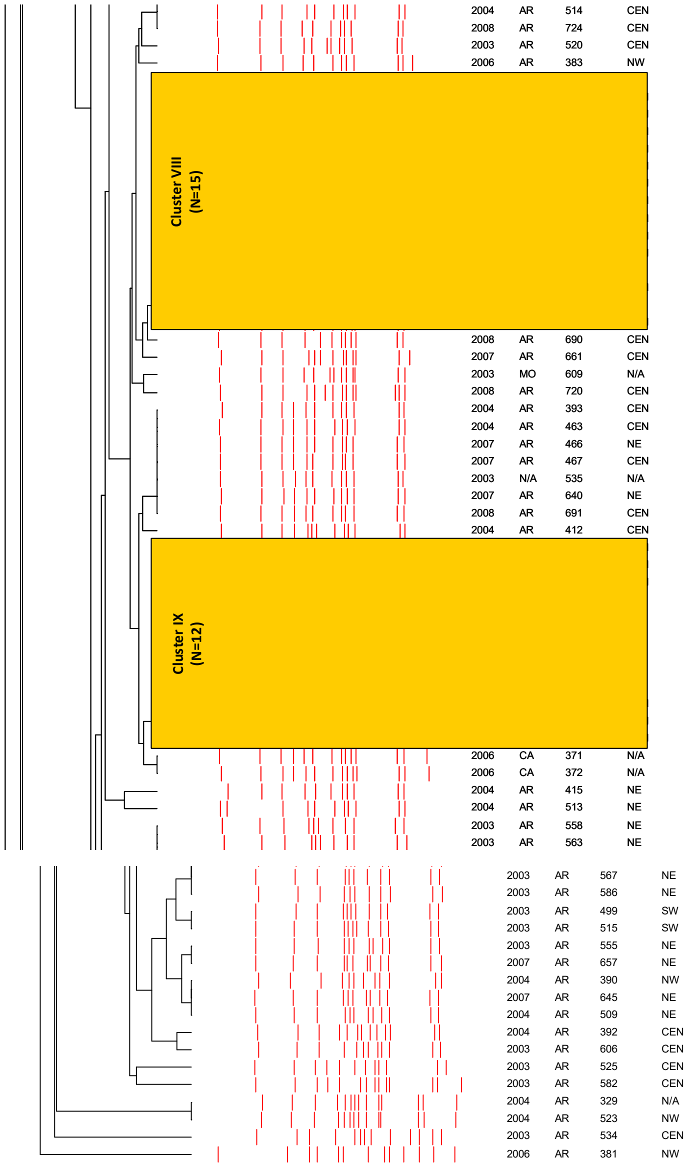
2004 AR 514 CEN
2008 AR 724 CEN
2003 AR 520 CEN
2006 AR 383 NW
Cluster VIII (N=15)
2008 AR 690 CEN
2007 AR 661 CEN
2003 MO 609 N/A
2008 AR 720 CEN
2004 AR 393 CEN
2004 AR 463 CEN
2007 AR 466 NE
2007 AR 467 CEN
2003 N/A 535 N/A
2007 AR 640 NE
2008 AR 691 CEN
2004 AR 412 CEN
Cluster IX (N=12)
2006 CA 371 N/A
2006 CA 372 N/A
2004 AR 415 NE
2004 AR 513 NE
2003 AR 558 NE
2003 AR 563 NE
2003 AR 567 NE
2003 AR 586 NE
2003 AR 499 SW
2003 AR 515 SW
2003 AR 555 NE
2007 AR 657 NE
2004 AR 390 NW
2007 AR 645 NE
2004 AR 509 NE
2004 AR 392 CEN
2003 AR 606 CEN
2003 AR 525 CEN
2003 AR 582 CEN
2004 AR 329 N/A
2004 AR 523 NW
2003 AR 534 CEN
2006 AR 381 NW
